# Characteristics and clinical outcomes of nirmatrelvir/ritonavir (Paxlovid^TM^) recipients in Canada, 2022: a descriptive cohort study

**DOI:** 10.14745/ccdr.v49i10a05

**Published:** 2023-10-01

**Authors:** Nadine Sicard, Susan Squires, Muhammad Mullah, Peter Daley

**Affiliations:** 1 Public Health Agency of Canada, Infectious Disease Programs Branch, Montréal, QC; 2 Public Health Agency of Canada, Infectious Disease Programs Branch, Ottawa, ON; 3 Public Heath Agency of Canada, Centre for Communicable Diseases and Infection Control, Ottawa, ON; 4 Memorial University of Newfoundland, Faculty of Medicine, Division of Infectious Diseases, St. John’s, NL

**Keywords:** nirmatrelvir/ritonavir, Paxlovid, antiviral therapy, COVID-19, health databases, provincial summary data

## Abstract

**Background:**

Nirmatrelvir/ritonavir (N/R) (Paxlovid^TM^) was introduced in Canada in January 2022. This was the first oral coronavirus disease 2019 (COVID-19) antiviral therapy that was deployed on a large scale in Canada. Since N/R was a new therapeutic option to reduce severe outcomes in high-risk populations, clinical and implementation questions were raised about its real-world utilization and impact. The objective of this retrospective observational study was to describe the characteristics and clinical outcomes of recipients of N/R in the first several months of its availability in Canada, during the Omicron wave.

**Methods:**

Provincial summary data were pooled together for the analysis. Descriptive statistics were used to explore the characteristics and clinical outcomes of the recipients. Pearson’s Chi-square test and unadjusted odds ratio along with 95% confidence intervals were used to identify the potential risk factors for severe outcomes. Data were generally collected between January and September 2022.

**Results:**

Seventy-six percent of N/R recipients were 60 years of age and older and 56% were female. Eighty-four percent of recipients had received three or more COVID-19 vaccinations and 67% had comorbidities. All-cause severe 30-day outcomes were uncommon, with 0.4% reported as deceased, 0.1% admitted to the intensive care unit and 2.0% hospitalized after N/R administration. Risk factors statistically associated with severe outcomes were immunosuppression, comorbidities, age of 60 years and older, and being unvaccinated.

**Conclusion:**

In the first months of its availability in Canada, N/R was mostly used in vaccinated patients 60 years and older with one or more comorbidities. Severe outcomes in N/R recipients were uncommon and mostly reported in patients with risk factors.

## Introduction

On January 17, 2022, nirmatrelvir/ritonavir (N/R or Paxlovid^TM^) was authorized by Health Canada ((1)) as the first oral antiviral coronavirus disease 2019 (COVID-19) treatment for adults who had mild to moderate symptoms, had positive severe acute respiratory syndrome coronavirus 2 viral test results, and were at high risk for progression to severe COVID-19, namely hospitalization or death. The regulatory approval was supported by the interim results of the manufacturer’s phase 2/3 randomized controlled trial, which assessed efficacy and safety in high-risk unvaccinated adults prior to the emergence of Omicron variants of concern. Participants in this study were eligible if they had at least one characteristic or coexisting condition associated with high risk of progression to severe COVID-19 (such as age of 65 years and older, smoking, diabetes, hypertension, immunosuppression, cardiovascular, pulmonary or kidney diseases, etc.) The results of this study demonstrated an 89% reduction in the composite outcome of COVID-19-related hospitalization or all-cause death from 6.4% to 0.78% (95% confidence interval [CI]: −7.21%–−4.03%) through a 28-day follow-up when treated within five days of symptom onset, compared to placebo. In subgroup analyses, the reduction of COVID-19-related hospitalizations or all-cause death was shown to be of lower magnitude in patients younger than 65 years old (−4.35, 95% CI: −5.91–−2.79) compared to those 65 years and older (−13.93, 95% CI: −20.07–−7.80) or in those with 0–1 comorbidities (−4.76, 95% CI: −6.36–−3.16) compared to those with 2–3 comorbidities (−8.96, 95% CI: −13.59–−4.32) ((2)).

Given the fast-changing nature of the COVID-19 pandemic, questions were raised about the applicability of these study results in vaccinated patients and with new variants (e.g. Omicron). In addition, safety considerations of N/R included drug-drug interactions with several medications commonly used to manage comorbidities that can be associated with increased risk of COVID-19 severity, thus potentially constraining the use of N/R in some patient groups.

With limited “real world” experience with N/R in the Canadian context of highly vaccinated populations and new variants, evaluation of N/R usage was considered a priority by several stakeholders. As such, in collaboration with representatives from provinces, territories, some federal departments and clinical experts, the Public Health Agency of Canada (PHAC) developed an evaluation framework, where one of the components of the evaluation aimed to document the characteristics of the recipients of N/R in Canada and their outcomes, which are reported here. At the time when this study was initiated, the effectiveness of N/R in vaccinated patients was unknown.

The Public Health Agency of Canada assumed a leadership role in this evaluation, which is consistent with two of its mandates: to respond to public health emergencies and to strengthen intergovernmental collaboration on public health and facilitate national approaches to public health policy and planning.

The objectives of this evaluation were to describe the characteristics and clinical outcomes at 30 days for recipients of N/R in the first several months of its availability in Canada.

## Methods

An evaluation framework was developed in collaboration with clinical experts and federal and provincial representatives at the onset of the N/R rollout to answer questions regarding the characteristics (demographics and risk factors) and 30-day outcomes of N/R recipients using a descriptive cohort design. A descriptive study design was selected given the variability of available information across jurisdictions and complexity of establishing a standardized denominator that would have been needed for a cohort study. Seven provinces and one federal department contributed aggregated data for this evaluation, representing 74% of the Canadian population.

### Sources of data and variables

A data dictionary defining variables was prepared and used by participating jurisdictions following consultations. Jurisdictions may have interpreted the definition of the variables according to the criteria generally used in their administrative healthcare data systems. Data collection methodologies varied by jurisdiction and, in several jurisdictions, over time. Jurisdictions used one of three methodologies to collect characteristic and outcome data for N/R recipients within their jurisdictions: primary data collection typically conducted by telephone or online questionnaires; secondary data collection from pre-existing health databases or chart reviews; or a combination of both methods. Most jurisdictions used health databases, such as drug benefit, immunization registry and hospital discharge information systems, to obtain the data. Questionnaires and chart reviews were sometimes used to obtain information about the clinical outcomes when not available in the information systems.

**Figure 1:**
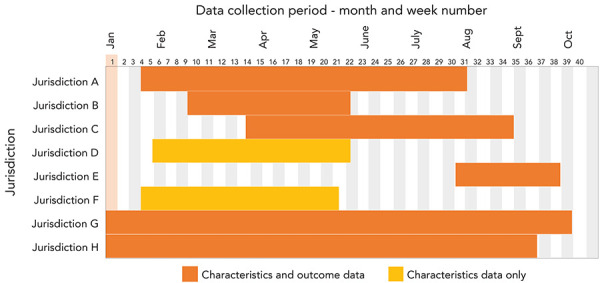
Data collection period by jurisdiction, evaluation of nirmatrelvir/ritonavir (Paxlovid^TM^) recipient characteristics and clinical outcomes, Canada^a^, 2022 ^a^ Based on data from eight reporting jurisdictions, with six reporting both characteristics and outcome data (solid orange bars) and two reporting only characteristics data (hashed orange bars)

Dates for data collection varied by jurisdiction depending on availability of information, but generally data were collected between January and September 2022. The availability and timeliness of information related to N/R recipient characteristics and clinical outcomes also varied by jurisdiction and within jurisdictions due to changes in the N/R programming over time, including eligibility criteria and accessibility. Six jurisdictions reported characteristics and outcome information, while two reported only characteristics information. The length of the data collection period ranged from nine to 39 weeks, with a median data collection period of 19 weeks. Not all jurisdictions were able to contribute data for all variables (**Figure 1**).

Outcomes were assessed at 30 days following the first day of N/R treatment. Severe outcomes, notably hospitalization, intensive care unit (ICU) admission and death, were measured as all-cause. Hospitalizations and death were chosen because one of the goals of the COVID-19 therapeutics response is to protect the population and the healthcare system by preventing hospitalizations and deaths. All-cause outcomes were used due to the feasibility of attributing COVID-19 cause in most participating jurisdictions’ data systems.

Participating jurisdictions used an aggregate summary table to report results and dates of collection. Summary tables from jurisdictions were submitted to PHAC between August 15, 2022, and November 8, 2022.

### Analyses

Summary table results from different jurisdictions were collated and analyzed by staff at the PHAC. Descriptive statistics were used to explore the characteristics and clinical outcomes of N/R recipients. To identify the potential risk factors for severe outcomes (hospitalized, admitted to ICU or death), Pearson’s Chi-square test was used to assess the association between each of the categorical variables and severe outcomes. Again, the unadjusted odds ratio (OR) along with the 95% CI were used to describe the direction and strength of the association between risk factors and severe outcomes. The OR represents the odds that an outcome will occur in one group compared to the odds of the outcome occurring in another group. Note that due to the lack of line list data, we could not calculate the adjusted ORs. Comparisons of jurisdictions were not conducted due to the variability in eligibility criteria to N/R and possible variations in data sources or definition. All statistical analyses were performed using R software (version 4.1.3).

## Results

### Characteristics nirmatrelvir/ritonavir recipients in Canada

**Figure 2:**
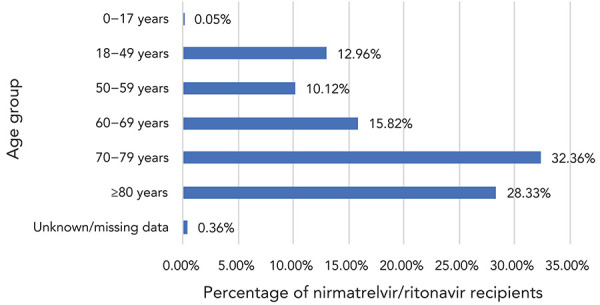
Age distribution of nirmatrelvir/ritonavir (Paxlovid^TM^) recipients, Canada^a^, 2022 (n=61,413) ^a^ Based on data from eight reporting jurisdictions

Age group and sex information were available for 61,413 patients: 77% of N/R recipients were 60 years of age and older, while 61% were 70 years of age and older (**Figure 2**). Fifty-six percent of N/R recipients were female.

**Figure 3:**
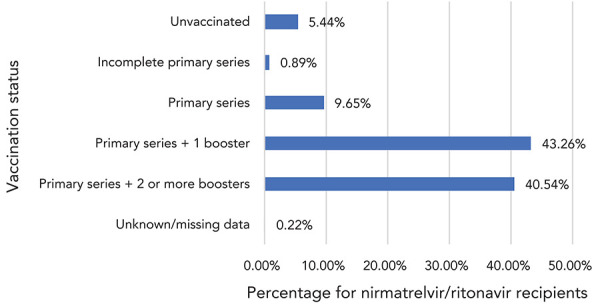
Distribution of vaccination status for nirmatrelvir/ritonavir (Paxlovid^TM^) recipients, Canada^a^, 2022 (n=59,452) ^a^ Based on data from six reporting jurisdictions. Primary series was defined as either two doses of a vaccine with a two-dose schedule (e.g. mRNA vaccines) or one dose of a vaccine with a one-dose primary series (e.g. Janssen Jcovden^®^ COVID-19 Vaccine). Incomplete primary series was defined as having received at least one COVID-19 vaccine but not meeting the requirements to have been considered as having completed the primary series, as per information in immunization records

**Figure 4:**
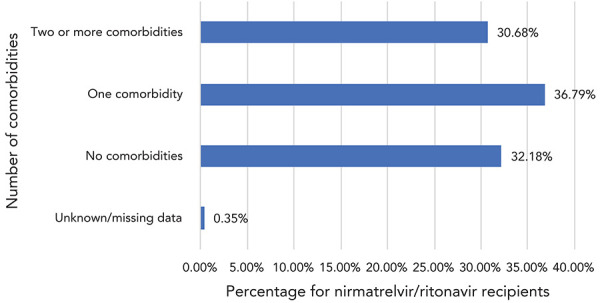
Distribution of the number of comorbidities in nirmatrelvir/ritonavir (Paxlovid^TM^) recipients, Canada^a,b^, 2022 (n=59,452) ^a^ Based on data from six reporting jurisdictions ^b^ “Comorbidities” refers to the following conditions: obesity (BMI≥30), chronic kidney disease, diabetes, heart disease, hypertension, congestive heart failure, chronic respiratory disease including cystic fibrosis, cerebral palsy, intellectual disability, sickle cell disease, moderate or severe kidney disease (eGFR<60 mL/min), and moderate or severe liver disease (e.g. Child Pugh, Class B or C cirrhosis)

Data on vaccination status (**Figure 3**) and number of comorbidities (**Figure 4**) were available for 59,452 N/R recipients across seven jurisdictions: 84% of recipients had received three or more COVID-19 vaccinations, while only 5% were unvaccinated.

Sixty-seven percent of recipients were identified as having one or more co-morbidities, while 5.8% were assessed as being immunocompromised. Data on additional factors associated with N/R administration were available for a subset of patients. Ninety-four percent (n=13,752/14,638) of recipients were symptomatic prior to COVID testing, and 95.5% (n=11,582/12,129) received N/R within five days of testing positive. Nine percent (n=1,689/19,196) of recipients received other COVID-19 therapeutics in addition to N/R.

Clinical outcomes at 30 days following first day of treatment with nirmatrelvir/ritonavir

**Figure 5:**
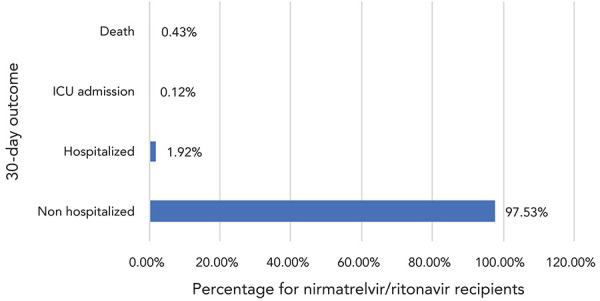
Distribution of 30-day outcomes for patients receiving nirmatrelvir/ritonavir (Paxlovid^TM^), Canada^a^, 2022 (n=58,881) Abbreviation: ICU, intensive care unit ^a^ Based on data from six reporting jurisdictions

Of the 58,881 recipients for whom outcome data were available, 97.5% were not hospitalized for any reason in the 30-day period post-N/R administration. All-cause severe outcomes were uncommon, with 0.4% (n=243) reported as deceased, 0.1% (n=67) admitted to the ICU and 2.0% hospitalized in the first 30 days after N/R administration (**Figure 5**).

**Table 1** presents a comparison of risk factors between subjects who experienced severe outcomes (hospitalized, admitted to ICU or death) and those who did not. The risk factors that showed a statistically significant association with severe outcomes (*p*<0.0001) include immunosuppressed status, vaccination status, age, sex and number of comorbidities. The odds/risk of having a severe outcome was significantly higher for patients who were immunocompromised (OR=3.79, 95% CI: 3.28–4.37, against non-immunocompromised), unvaccinated or partially vaccinated (OR=1.91, 95% CI: 1.62–2.26, against completed primary series or completed primary series with one or more booster dose), older age (60 years and older) (OR=1.64, 95% CI: 1.42–1.89, against age younger than 60 years), males (OR=1.25, 95% CI: 1.12–1.39, against females), and with one or more comorbidities (OR=1.97, 95% CI: 1.73–2.24, against no comorbidity).

**Table 1:** Risk factors associated with severe outcomes for patients dispensed nirmatrelvir/ritonavir (Paxlovid^TM^), Canada^a^, 2022

Risk factor	Percentage with severe outcomes	Odds ratio (95% CI)	*p*-value^b^
**Immunosuppressed status**			
Immunosuppressed	7.78	3.79 (3.28–4.37)	<0.0001
Not immunosuppressed	2.18	Reference	
**Vaccination status**			
Unvaccinated/incomplete primary series	4.37	1.91 (1.62–2.26)	<0.000
Primary series/primary series with one or more booster	2.33	Reference	
**Age**			
60 years and older	2.69	1.64 (1.42–1.89)	<0.0001
0–59 years	1.64	Reference	
**Sex**			
Male	2.73	1.25 (1.12–1.39)	<0.0001
Female	2.20	Reference	
**Number of comorbidities**			
One or more	2.92	1.97 (1.73–2.24)	<0.0001
None	1.50	Reference	

^a^ Based on data from six reporting jurisdictions

^b^ The *p*-values were obtained from Pearson’s Chi-squared test. Primary series was defined as either two doses of a vaccine with a two-dose schedule (e.g. mRNA vaccines) or one dose of a vaccine with a one-dose primary series (e.g. Janssen Jcovden^®^ COVID-19 Vaccine). Incomplete primary series was defined as having received at least one COVID-19 vaccine but not meeting the requirements for being considered having completed the primary series as per information in immunization records. The term “immunosuppressed” was defined as individuals meeting the following criteria: receipt of treatment for solid tumours and hematologic malignancies (including individuals with lymphoid malignancies who are being monitored without active treatment); receipt of solid-organ transplant and taking immunosuppressive therapy; receipt of chimeric antigen receptor (CAR)-T-cell or hematopoietic stem cell transplant (within two years of transplantation or taking immunosuppressive therapy); moderate or severe primary immunodeficiency (e.g. DiGeorge syndrome, Wiskott-Aldrich syndrome, common variable immunodeficiency, Good’s syndrome, hyper IgE syndrome); advanced or untreated HIV infection; and/or active treatment with high-dose corticosteroids (i.e. 20 mg or more prednisone or equivalent per day when administered for two or more weeks), alkylating agents, antimetabolites, transplant-related immunosuppressive drugs, cancer chemotherapeutic agents classified as severely immunosuppressive, tumour-necrosis factor (TFN) blockers, and other biological agents that are immunosuppressive or immunomodulatory

## Discussion

In the first months of its availability in Canada, the results of this descriptive cohort showed that N/R was mostly used in vaccinated patients (83.80% had received three or more doses) and patients over 60 years of age (76.51%); 30.68% were reported as having two or more comorbidities. In contrast, the study participants included in the phase 2/3 randomized controlled trial that supported the regulatory approval were unvaccinated, had a median age of 46 years and 61% had two or more characteristics or coexisting conditions placing them at high risk of progression to severe disease ((2)). The definitions of coexisting conditions and the methodology used for data collection differed in both studies making direct comparisons of the characteristics of the study populations difficult.

This study estimated that 2.5% of N/R recipients progressed to develop all-cause severe outcomes (hospitalization, ICU admission or death). A 0.4% all-cause mortality was also seen in N/R recipients. These rates of severe outcomes are slightly higher than in other published studies that reported severe outcome rates of less than 1% ((1,3,4)) or death rates of less than 0.4% ((4–6)). As well, a recent retrospective cohort study in the United States observed a 0.47% COVID-19-related hospitalization rate and a 0.01% mortality rate among recipients of N/R ((7))—results that were also lower than those observed in this study. A recent study from Ontario observed a 2.1% risk of hospitalization for COVID-19 or death at 30 days for patients treated with N/R ((8)).

These differences may be explained by the methodology used in our evaluation: we measured all-cause outcomes, while other studies measured COVID-19-specific outcomes. Additionally, recipients of N/R in Canada tended to be older ((1,5,6)) than other study populations, which may account for those studies’ lower severity rates. Many studies also followed patients for longer periods of time than the 30-day period in this evaluation or assessed COVID-19-specific severe outcomes, which made comparisons to this evaluation difficult. Furthermore, other studies used case-control study designs with various methods to control bias whereas this study was a descriptive cohort.

In this evaluation, severe outcomes were relatively rare and were highest in the following groups: the immunocompromised; the unvaccinated/partially vaccinated; those having one or more comorbidities; those aged 60 years or older; and males. These results are consistent with those who have been identified as being most at risk for severe outcomes ((9)) and thus would benefit the most from N/R. Since this evaluation did not include a comparison group, the evaluation was unable to determine benefit of N/R in preventing higher rates of severe outcomes.

## Limitations

This evaluation has several limitations. Characteristics and clinical outcomes of N/R recipients were assessed in jurisdictions that self-selected to participate in this evaluation. As only aggregate level data were collected, it was not possible to conduct sub-analyses nor assess for any confounding or interaction effects among the characteristics with respect to the outcomes. Data from multiple jurisdictions were aggregated to form a national dataset although there were variations in the distribution of clinical outcomes by patients’ characteristics across the different jurisdictions. We could not account for the dissimilarities in the data from each jurisdiction. Data collection periods and eligibility criteria varied by jurisdiction and by time, which may have impacted the description of the characteristics. For example, early on when N/R supply was limited and criteria were stricter, patients may have been older and at higher risk for severe outcomes. Participation by jurisdictions was voluntary. Although the jurisdictions that did participate represented a significant percentage of the Canadian population (74%), this evaluation may not be fully representative of all N/R recipients in Canada. It is well established that COVID-19 disproportionately affects racialized and marginalized populations ((10)); however, as ethnicity data were not available for this evaluation, the impact of ethnicity on outcomes among N/R recipients was not assessed. Finally, the results of this evaluation may have underestimated the proportion of patients who did not experience a severe outcome as it is possible that some patients were included who may never have started or may not have completed treatment but were deemed “N/R recipients.”

## Conclusion

Despite its limitations, these findings were useful in providing an assessment of who has received N/R in the first months of its availability in Canada and provided information about the low rate of severe outcomes in mostly vaccinated N/R recipients at the national level. While awaiting results from adaptive platform trials or other randomized controlled studies on the real-world effectiveness of N/R, which will require more time to perform, this evaluation provided participating jurisdictions with useful information to inform decision-making with respect to N/R programming and policies in the interim. As more COVID-19 therapeutics are developed and become available, similar questions may arise. The collaborative model used for this evaluation project could be employed in the future to answer similar questions with other therapies; however, it should preferably strive to include a comparison group in the methodology, which would enable adjusting for confounders and assessing effectiveness.
